# Elevated Risk of Carrying Gentamicin-Resistant *Escherichia coli* among U.S. Poultry Workers

**DOI:** 10.1289/ehp.10191

**Published:** 2007-09-04

**Authors:** Lance B. Price, Jay P. Graham, Leila G. Lackey, Amira Roess, Rocio Vailes, Ellen Silbergeld

**Affiliations:** 1 The Johns Hopkins University School of Medicine, Baltimore, Maryland, USA; 2 The Johns Hopkins University Bloomberg School of Public Health, Baltimore, Maryland, USA

**Keywords:** antibiotic, antimicrobial, aminoglycoside, chickens, *Escherichia coli*, gentamicin, occupational exposure, poultry, resistance, worker

## Abstract

**Background:**

Antimicrobial use in food-animal production is an issue of growing concern. The application of antimicrobials for therapy, prophylaxis, and growth promotion in broiler chicken production has been associated with the emergence and dissemination of antimicrobial-resistant enteric bacteria. Although human exposure to antimicrobial-resistant bacteria through food has been examined extensively, little attention has been paid to occupational and environmental pathways of exposure.

**Objective:**

Our objective was to measure the relative risk for colonization with antimicrobial-resistant *Escherichia coli* among poultry workers compared with community referents.

**Methods:**

We collected stool samples and health surveys from 16 poultry workers and 33 community referents in the Delmarva region of Maryland and Virginia. *E. coli* was cultured from stool samples, and susceptibility to ampicillin, ciprofloxacin, ceftriaxone, gentamicin, nitrofurantoin, and tetracycline was determined for each *E. coli* isolate. We estimated the relative risk for carrying antimicrobial-resistant *E. coli* among poultry workers compared with community referents.

**Results:**

Poultry workers had 32 times the odds of carrying gentamicin-resistant *E. coli* compared with community referents. The poultry workers were also at significantly increased risk of carrying multidrug-resistant *E. coli*.

**Conclusions:**

Occupational exposure to antimicrobial-resistant *E. coli* from live-animal contact in the broiler chicken industry may be an important route of entry for antimicrobial-resistant *E. coli* into the community.

Antimicrobial-resistant bacteria including *Escherichia coli* are common contaminants of the industrial broiler chicken environment (Hayes et al. 2004; Khan et al. 2005). Studies conducted in Europe indicate that poultry growers and poultry-house workers are at risk of exposure to these and other pathogens (Ojeniyi 1989; van den Bogaard et al. 2001). Similar studies must be conducted in the United States in order to measure occupational exposure to antimicrobial-resistant *E. coli* in the U.S. broiler chicken industry.

Antimicrobial use has been integral to the industrialization of food-animal production. Over the past half century, food-animal production has changed from a largely entrepreneurial system run by independent farmers to an industrial mode of production in which a small number of companies control all aspects of production, from breeding and feed formulation to slaughter and distribution of consumer products. This shift in both the organization and methods of production has allowed for the reliable, high-throughput production of food animals on a scale not seen in human history. In the United States alone, > 9 billion food animals are produced annually (U.S. Department of Agriculture 2004). Antimicrobials have been used in food-animal production since the early days of their discovery (Viola and DeVincent 2006). Although exact figures are unavailable, it is currently estimated that at least half of all of the antimicrobials consumed in the United States are used in food-animal production (Steinfeld et al. 2006). Antimicrobials are used for multiple purposes including outbreak control, prophylaxis, and growth promotion. The antimicrobials approved for use in the U.S. food-animal industry include many drugs of critical importance to human medicine. Sixteen antimicrobial agents from 10 antimicrobial classes are currently approved for use in U.S. poultry production (Appendix 1). Among these, gentamicin (GEN) is reported to be the most commonly used antimicrobial (Luangtongkum et al. 2006).

The use of antimicrobials in industrial food-animal production selects for antimicrobial-resistant bacterial populations. Antimicrobial-resistant bacteria have been detected in animal wastes (Jindal et al. 2006), animal bedding (Kelley et al. 1998), air both inside (Chapin et al. 2005) and downwind (Gibbs et al. 2006) of animal feeding operations, in groundwater near animal feeding operations (Anderson and Sobsey 2006), and in consumer meat and poultry products (Food and Drug Administration 2006). There is substantial evidence that antimicrobial use in food-animal production contributes to the burden of antimicrobial-resistant diseases in human populations through foodborne routes of exposure. Examples include infections with fluoro-quinolone-resistant *Campylobacter* (Gupta et al. 2004)*,* vancomycin-resistant *Enterococci* (VRE) (Bonten et al. 2001), multidrug-resistant *Salmonella* (Molbak 2006), and multidrug-resistant *E. coli* (Ramchandani et al. 2005). Additional pathways likely exist through exposure to contaminated environmental media in and around animal production facilities as well as contact with animals in the occupational setting.

Some of the seminal reports connecting antimicrobial use in food-animal production with antimicrobial-resistant human infections and colonization involved studies of farmers and their families (Levy 1978; Levy et al. 1976). These early reports have been confirmed by more recent case studies (Fey et al. 2000; Oppegaard et al. 2001). Occupational epidemiology studies of European broiler farmers and turkey farmers, as well as broiler and turkey slaughterhouse workers, indicated that these populations were at increased risk of colonization with antimicrobial-resistant *E. coli* and *Enterococcus* because of their occupational exposure to these animals (Stobberingh et al. 1999; van den Bogaard et al. 1997, 2001, 2002). To date, no studies have been published assessing the occupational exposures to antimicrobial-resistant enteric bacteria incurred by workers in the U.S. broiler industry.

The U.S. broiler industry is estimated to employ > 200,000 people (U.S. Poultry and Egg Association 2006), and the industrialization of production has resulted in the division of labor into specific tasks. Most broiler houses are owned and operated by independent farmers called “growers.” Growers typically raise chickens under contract with production companies or integrators. At the end of the grow-out period, chickens are captured by people called “catchers,” placed into cages, and transported to the slaughtering facilities. Once delivered to the slaughterhouse, the broilers are removed from the cages and shackled to semiautomated slaughter lines by people called “live hangers.” Thus, growing, catching, and hanging are the three tasks with the most intensive live-animal exposures. For example, a catcher will capture and cage thousands of chickens in a single workday (Goodman 2006). Defining risks for these highly exposed populations may have relevance to understanding the dissemination of antimicrobial-resistant bacteria more generally within communities.

In the present study, we compared the risk of antimicrobial-resistant *E. coli* colonization among poultry workers (i.e., growers, catchers, and live hangers) and rural community referents. We cultured stool samples for *E. coli* and determined the susceptibility of isolates to six different antimicrobials. We estimated the relative risk for colonization with antimicrobial-resistant *E. coli* among poultry workers compared to referents.

## Materials and Methods

The present study was designed as a convenience sample of poultry workers and community residents in the eastern shore region of Maryland and Virginia. The study complied with all U.S. regulations regarding research on human subjects and was approved by the Johns Hopkins Medical Institutions Committee on Human Subjects Research. All human subjects provided written informed consent prior to participating in the study.

### Study site and enrollment

Subjects were recruited and enrolled 29 April–1 May 2005 and 4–5 December 2005 at several sites in southern Maryland and Virginia on the Delmarva Peninsula. This region is among the top five broiler chicken regions in the United States, producing > 600 million chickens annually. Subjects were recruited and enrolled into the study using a convenience-sampling approach, leveraging public notices (fliers and newspaper advertisements), personal outreach through a local nongovernmental organization (Delmarva Poultry Justice Alliance), and door-to-door inquiries in neighborhoods known to be populated by poultry workers. The study excluded anyone < 18 years of age, those employed in the medical industry, those working in a poultry processing plant, and those who had traveled outside the United States in the past 3 months. A modest incentive ($25 gift certificate) was offered to those willing to complete a questionnaire and provide stool samples for analysis.

### Questionnaire

After meeting the minimum inclusion criteria for the overall health study, subjects were administered a face-to-face questionnaire consisting of three parts. Part 1 covered demographic information including sex, race, and level of education; employment status within the past 6 months; health insurance status; and primary source of health care (i.e., doctor’s office, hospital, workplace, or other). Part 2 included nonoccupational risks for exposure to antimicrobial-resistant *E. coli* from animal sources such as well water, ground red meat, and poultry consumption, and the presence of pets in the household. Part 3 was directed to those reporting recent or current employment in the broiler chicken industry, and requested information on job title (catcher, grower, or live hanger), duration of employment, average frequency of exposure over the course of a week, and days worked in the week immediately before study participation. Subjects were also asked about availability and use of personal protective equipment. Finally, subjects were asked whether clothing worn at work was laundered at home. All data were collected under complete confidentiality; after enrollment, each subject was identified solely by an alphanumeric code.

### Stool collection and culture

Participants were provided a stool sample collection kit (Fisher Scientific, Pittsburgh, PA) and were asked to produce and collect a sample on site or to take the kit home and return a sample within 4 hr of collection. Stool samples were transferred to Enteric Plus transport media (Meridian Bioscience, Cincinnati, OH) (Wasfy et al. 1995) and kept at 4°C until they were cultured in the laboratory within 72 hr of arrival. *Enterobacteriaceae* was cultured from the transport media on standard Violet Red Bile Glucose Agar (Oxoid, Hampshire, UK) and on Violet Red Bile Glucose Agar supplemented with one each of the following antimicrobials: ampicillin (AMP), 16 μg/mL; ciprofloxacin (CIP), 2 μg/mL; ceftriaxone (CEF), 32 μg/mL; GEN, 8 μg/mL; and tetra-cycline (TET), 8 μg/mL (i.e., each sample was cultured on six different media formulations). Isolates were purified twice on the same medium on which they were isolated. All isolates were stored at −80°C until they were tested for antimicrobial susceptibility as described below. Selective antimicrobials were used to increase the sensitivity of the assay for detecting resistant *E. coli* strains among a mix of resistant and susceptible strains within the stool sample.

### Susceptibility testing

Minimal inhibitory concentrations were determined by the agar dilution method using *E. coli* ATCC 25922 and *E. coli* ATCC 35218 (both from American Type Culture Collection, Manassas, VA) as reference strains as recommended by guidelines of the Clinical and Laboratory Standards Institute (2002). The dilution ranges (resistance breakpoints) were as follows: AMP, 0.25–32 (32); CIP, 0.004–8 (4); CEF, 0.06–64 (64); GEN, 0.25–16 (16); NIT, 2–256 (128); and TET, 1–16 (16).

### Species identification

Isolates were identified to the species level using the Microbact 12A (12E) Gram-Negative Identification System—Strip Format (Oxoid). Following the protocol provided by the manufacturer, isolates were cultured for 24 hr on Mueller-Hinton blood agar, confirmed oxidase-negative, suspended in a saline solution, and incubated for 24 hr in a test strip containing 12 distinct substrates commonly used for *Enterobacteriaceae* species identification. Strips were scored based on color change, and results were correlated with genus and species using the Microbact 2000 Identification Package, V2.03 for Windows (Oxoid). Only those isolates positively identified as *E. coli* were used for outcome analyses.

### Sample scoring

Stool samples were plated on six different agars as described above; therefore, up to six isolates were cultured from each participant (i.e., one isolate per agar formulation). If ≥1 of the *E. coli* isolates from a participant’s stool sample was resistant to an antimicrobial, the sample was scored as resistant. If any single isolate from a participant’s stool culture was resistant to ≥2 antimicrobials, the sample was scored as multidrug resistant. In total, eight different antimicrobial resistance outcomes were measured: AMP resistance, CIP resistance, CEF resistance, GEN resistance, NIT resistance, TET resistance, antimicrobial resistance (any of the six), and multidrug resistance (≥2).

### Data entry

Questionnaire data were entered into a database using EpiData 3.1 (Lauritsen and Bruus 2005) by two different researchers. Referencing the original questionnaires allowed for inconsistencies between the independent entries to be rectified.

### Statistical analyses

We used odds ratios, Fisher’s exact tests, and chi-square analyses to assess the associations between exposure (poultry worker vs. community reference) and the eight outcomes. Bivariate logistic regression was used to measure these associations while adjusting for self-reported antibiotic use during the month before sample collection. All statistical analyses were performed using Stata 8.0 (StataCorp, College Station, TX).

## Results

Seventy-one people were enrolled into our study, but 49 subjects were included in the analyses presented here. Nine people were excluded because they shared a residence with a poultry worker, and 13 were excluded because *E. coli* was not recovered from their stool samples. There was not a significant difference in the proportion of poultry workers and community referents dropped for lack of an *E. coli* culture; therefore, their exclusion did not affect the internal validity of the study.

Poultry workers (*n* = 16) and community referents (*n* = 33) were similar with respect to most of the general characteristics assessed in the study (Table 1). There were fewer poultry workers who reported completing high school than community referents, but we did not consider this to be a potential confounder for the analyses presented here.

Most nonoccupational risk factors for colonization with antimicrobial-resistant *E. coli* were similar between the two exposure groups. Poultry workers and community referents were similar with respect to self-reported exposures to potential sources of animal-associated antimicrobial-resistant *E. coli*, including ground red meat, poultry, and well-water consumption, as well as keeping pets (Table 2). The two groups were also similar in the proportion of participants who sought health care in the month before the study. However, poultry workers were more than two times as likely as referents to report antibiotic use during this same period (Table 2). Self-reported antibiotic use was associated with increased odds for all eight outcomes of interest (Table 3) and therefore was included in multivariate logistic regression models.

Poultry workers had 32 times the odds of being colonized with GEN-resistant *E. coli* compared with community referents (Table 4). AMP-resistant and TET-resistant *E. coli* carriage was common among both groups and not significantly different between them (Table 4). In contrast, CIP-, CEF-, and NIT-resistant *E. coli* were uncommon in both groups— too rare to calculate relative risk estimates. Nine different multidrug-resistance patterns were identified among the *E. coli* strains collected (Appendix 2). Carriage of multidrug-resistant *E. coli* was significantly more common in poultry workers and was largely attributable to the disproportionate prevalence of GEN resistance (Table 4). Adjusting for self-reported antibiotic use in multivariate logistic analyses did not change the overall trends and had little effect on the individual associations (Table 4).

## Discussion

This is the first U.S.-based study to assess the risk for colonization with antimicrobial-resistant *E. coli* from occupational exposure to live chickens in the broiler chicken industry. The results presented here confirm similar studies in Europe showing that poultry farmers and slaughterhouse workers were at excess risk for colonization with antimicrobial-resistant *E. coli* (van den Bogaard et al. 2001).

In the present study, 50% of the poultry workers were colonized with GEN-resistant *E. coli*. This was in stark contrast to the proportion of community referents colonized with GEN-resistant *E. coli* (3%) and to the rates reported among hospital isolates (6.3%) (Friedland et al. 2003). Aminoglycosides are not absorbed through the gastrointestinal tract; therefore, human use is limited to intravenous, intramuscular, subcutaneous, and topical administration. The inability to administer GEN orally limits outpatient use; thus, there is probably minimal selection of GEN-resistant *E. coli* in the community. In contrast, GEN has been reported to be the most commonly used antimicrobial in broiler production, where it is administered prophylactically to day-old chicks to prevent bacterial infections (Luangtongkum et al. 2006). GEN resistance is common among poultry-associated enteric bacteria. A recent survey revealed that 69% of avian pathogenic *E. coli* isolates were resistant to GEN (Zhao et al. 2005). The disproportionately high prevalence of GEN-resistant *E. coli* among the poultry workers in the present study is consistent with the hypothesis that poultry workers are at increased risk of becoming colonized with antimicrobial-resistant *E. coli* resulting from occupational exposures to these strains in the broiler chicken environment.

We acknowledge the limitations of the present study, particularly the small sample size. The study design was a community-based convenience sample necessitated by the fact that poultry workers in contact with live poultry are unorganized and often contracted by integrators. Thus, there was no reliable way to recruit subjects through labor organizations or employers. The sample size was further limited by our inability to culture *E. coli* from the stools of 13 participants. Culture recovery might have been improved by using a type of medium more selective for *E. coli*. Stool sample cultivability may also have been reduced because of variable delays between the time participants collected their stool samples and the time they delivered them to researchers (participants were requested to do so within 4 hr). There was not a significant difference in the proportion of poultry workers and community referents dropped from the analysis due to negative cultures; therefore, the internal validity of the study was not affected by this limitation.

We did not ascertain health outcomes that may have been related to antimicrobial-resistant *E. coli* colonization, but the increased colonization rates among poultry workers may be associated with increased health risks in this population. Poultry-associated multidrug-resistant strains of *E. coli* have been shown to cause urinary tract infections and sepsis in the community (Manges et al. 2006; Ramchandani et al. 2005). Furthermore, antibiotic-resistant *E. coli* can serve as a reservoir of mobile resistance determinants that can be transferred to pathogenic bacterial species (Blake et al. 2003). Thus, harboring *E. coli* with these determinants may increase one’s risk for developing an antimicrobial-resistant infection.

Colonization with antimicrobial-resistant *E. coli* may also pose a health risk to poultry workers’ families and community contacts. Community-based studies indicate that strains of antimicrobial-resistant *E. coli* are often shared among family members and between domestic partners (Hannah et al. 2005; Lietzau et al. 2006). In addition to the fecal–oral route, poultry workers’ household contacts may be exposed to antimicrobial-resistant *E. coli* from contaminated work clothes (88% of poultry workers in the present study reported laundering their work clothes at home; data not shown).

To determine whether colonization by *E. coli* is transient or long-term among poultry workers, prospective studies complemented by detailed genetic analysis will be required. Likewise, such analyses can reveal whether mobile resistance elements are transferring resistance to native microbial flora in poultry workers. Additional studies will also be necessary to evaluate the potential for negative health outcomes associated with colonization by antimicrobial-resistant *E. coli* and to determine risks of exposure for household contacts of poultry workers.

A number of U.S. poultry producers have announced that they have substantially reduced their antimicrobial consumption by discontinuing the use of antimicrobial growth promoters (antimicrobials added to feed to promote growth) (Weise 2006). GEN is not approved for growth promotion in the United States; therefore, consumption rates will be unaffected by decreases in antimicrobial growth promoters. The decision by Tyson Foods (Springdale, AR), one of the nation’s largest poultry producers, to stop using antibiotics altogether for chickens marketed fresh in the United States could result in a substantial reduction in GEN use; however, this change will be implemented at less than half of their U.S. production facilities (Associated Press 2007). Furthermore, it is unclear whether this new antimicrobial use policy will be implemented at Tyson’s foreign production plants.

## Conclusion

Occupational exposure to live animals in the broiler chicken industry may be an important route of entry for antimicrobial-resistant bacteria into the community. The present study revealed a disproportionately high rate of colonization with GEN-resistant *E. coli* among U.S. poultry workers. Although there have been reports of decreased antimicrobial consumption by some U.S. poultry producers, antimicrobial use is still common in broiler chicken production in the United States and around the world. The continued use of GEN for food-animal production threatens the clinical utility of this and other aminoglycosides for human medicine.

## Figures and Tables

**Table 1:** Participant characteristics [percent (no.)] for community referents (CR; *n* = 33) and poultry workers (PW; *n* = 16).

Characteristic	CR	PW
Age [years (mean ± SD)]	42.5 ± 13.1	46.4 ± 8.3
Sex		
Male	69.7 (23)	88.9 (16)
Female	30.3 (10)	11.1 (2)
Race
White	12.1 (4)	16.7 (3)
Black	87.9 (29)	77.8 (14)
Other	0 (0)	5.5 (1)
Education		
< High school	39.4 (13)	61.1 (11)
High school*	45.5 (15)	16.7 (3)
> High school	15.1 (5)	22.2 (4)
Health insurance^a^		
Yes	60.6 (20)	72.2 (13)
No	39.4 (13)	27.8 (5)

a Health insurance data were missing for one CR.

* Significantly different between exposure groups (*p* < 0.05).

**Table 2:** Nonoccupational risk factors [percent (no.)] for antimicrobial-resistant *E. coli* carriage exposure in community referents (CR; *n* = 33) and poultry workers (PW; *n* = 16).

Risk factor	CR	PW
Antibiotic use previous month		
Yes	18.2 (6)	33.3 (6)
No	81.8 (27)	66.7 (12)
Health care in previous month		
Yes	15.2 (5)	12.5 (2)
No	84.8 (28)	87.5 (14)
Consume ground red meat		
Yes	93.9 (31)	100.0 (18)
No	6.1 (2)	0 (0)
Consume poultry		
Yes	100.0 (33)	100.0 (18)
No	0 (0)	0 (0)
Own pets		
Yes	33.3 (11)	12.5 (2)
No	66.7 (22)	87.5 (14)
Drinking water source		
In/out well	33.3 (11)	38.9 (7)
Public water	36.4 (12)	44.4 (8)
Bottled water	27.3 (9)	11.1 (2)
Unknown	3.0 (1)	5.5 (1)

**Table 3:** Odds of antimicrobial-resistant *E. coli* among participants reporting any antibiotic use during the month before the study.

	Antimicrobial-resistant cases		Antimicrobial user/nonuser	
Resistance	Users (*n* = 12) [Percent (no.)]	Nonusers (*n* = 27) [Percent (no.)]	OR (95% CI)	*p-*Value
AMP^R^	83.3 (10)	54.1 (20)	4.25 (0.73–44.0)	0.071
CIP^R^	16.7 (2)	2.7 (1)	7.20 (0.33–436)	0.080
CEF^R^	0.0 (0)	2.7 (1)	—	0.565
GEN^R^	25.0 (3)	16.2 (6)	1.72 (0.23–10.1)	0.495
NIT^R^	0.0 (0)	2.7 (1)	—	0.565
TET^R^	50.0 (6)	37.8 (14)	1.64 (0.36–7.47)	0.456
R	91.7 (11)	67.6 (25)	5.28 (0.61–246)	0.100
MDR	58.3 (7)	29.7 (11)	3.31 (0.71–16.0)	0.074

Abbreviations: CI, confidence interval; MDR, multidrug resistant; OR, odds ratio; R, resistant to ≥1 antimicrobials (superscript indicates resistance to a specific antimicrobial).

**Table 4:** Odds of antimicrobial-resistant *E. coli* among poultry workers and community referents.

	Antimicrobial- resistant cases		PW/CR (unadjusted)		PW/CR (adjusted)^a^	
Resistance	PW % (no.)	CR % (no.)	OR (95% CI)	*p-*Value	OR (95% CI)	*p-*Value
AMP^R^	62.5 (10)	60.6 (20)	1.08 (0.27–4.56)	0.899	0.83 (0.22–3.10)	0.786
CIP^R^	6.25 (1)	6.06 (2)	1.03 (0.02–21.3)	0.979	0.63 (0.05–8.74)	0.730
CEF^R^	6.25 (1)	0.00 (0)	—	0.147	—	—
GEN^R^	50.0 (8)	3.03 (1)	32.0 (3.21–1,462)	< 0.001	33.1 (3.47–316)	0.002
NIT^R^	6.25 (1)	0.00 (0)	—	0.147	—	—
TET^R^	56.3 (9)	33.3 (11)	2.57 (0.64–10.5)	0.126	2.43 (0.70–8.48)	0.163
R	81.3 (13)	69.7 (23)	1.88 (0.38–12.4)	0.390	1.52 (0.34–6.90)	0.584
MDR	62.5 (10)	24.2 (8)	5.21 (1.21–23.1)	0.009	4.61 (1.23–17.2)	0.023

Abbreviations: CI, confidence interval; CR, community referent; MDR, multidrug resistant; PW, poultry worker; R, resistant to ≥ 1 antimicrobials (superscript indicates resistance to a specific antimicrobial).

a Based on logistic regression model adjusting for self-reported use of any antibiotics during the month before sample collection.

## Appendix 1. Antimicrobial agents approved for use in broiler production (adapted from the National Research Council 1999)

**Table:**

Antimicrobial class	Agent
Aminocoumarin	Novobiocin
Aminocyclitols	Spectinomycin^a^
Aminoglycosides	Streptomycin
	Neomycin
	Gentamicin
β-Lactams	Penicillin^a^
Decapeptides	Bacitracin^a^
Lincosamides	Lincomycin
Macrolides	Erythromycin
	Tylosin^a^
	Oleandomycin^a^
Organoarsenicals	Roxarsone
Phosphoglycolipids	Bambermycin^a^
Streptogramins	Virginiamycin^a^
Tetracyclines	Chlortetracycline^a^
	Oxytetracycline
	Tetracycline

a Labeled as growth promoter

## Appendix 2. Observed multidrug-resistance patterns in *E. coli* strains

**Table:**

AMP^R^	CIP^R^	CEF^R^	GEN^R^	NIT^R^	TET^R^	No. of strains
x					x	1
x		x				1
x	x					1
x					x	4
			x		x	2
x					x	3
			x		x	4
x	x				x	1
x			x	x		1

R, resistant to that specific antimicrobial.
